# High-performance therapeutic quercetin-doped adhesive for adhesive–dentin interfaces

**DOI:** 10.1038/s41598-017-08633-3

**Published:** 2017-08-15

**Authors:** Hongye Yang, Kang Li, Huiyi Yan, Siying Liu, Yake Wang, Cui Huang

**Affiliations:** The State Key Laboratory Breeding Base of Basic Science of Stomatology (Hubei-MOST) & Key Laboratory for Oral Biomedical Ministry of Education, School & Hospital of Stomatology, Wuhan University, Wuhan, People’s Republic of China

## Abstract

Almost half of dental restorations have failed in less than 10 years, and approximately 60% of practice time has been consumed to replace these dental restorations. As such, contemporary dentin adhesives should be modified to treat secondary caries and prevent the degradation of adhesive–dentin interfaces. To achieve this goal, we developed a versatile therapeutic adhesive in the present study by incorporating quercetin, which is a naturally derived plant extract, into a commercial adhesive at three concentrations (100, 500 and 1000 µg/mL). An unmodified adhesive served as a control. The antibacterial ability on *Streptococcus mutans* biofilm, conversion degree, microtensile bond strength, failure modes, *in situ* zymography, nanoleakage expression and cytotoxicity of quercetin-doped adhesive were comprehensively evaluated. Results showed that the quercetin-doped adhesive (500 µg/mL) preserved its bonding properties against collagenase ageing and inhibited the growth of *S. mutans* biofilm. Efficient bonding interface sealing ability, matrix metalloproteinase inhibition and acceptable biocompatibility were also achieved. Thus, a simple, safe and workable strategy was successfully developed to produce therapeutic adhesives for the extension of the service life of adhesive restorations.

## Introduction

Dental composites are commonly used in daily oral clinical settings because of their excellent aesthetic performance^1, 2^. However, almost half of dental restorations have failed in less than 10 years, and they took dentists about 60% of practice time to replace^3–5^. Composite restorations are bonded to tooth structure via adhesives^6, 7^, and this replacement is attributed to secondary caries at bonding interfaces and adhesives’ poor mechanical strength induced by degradation^8, 9^. Thus, the longevity of adhesive restorations should be improved by incorporating bioactive agents to treat recurrent caries and prevent the degradation of adhesive–dentin interfaces^10, 11^.

Residual or invading acidogenic bacteria, such as *Streptococcus mutans*, can exist along the adhesive–dentin interface^12, 13^. To avoid cariogenic bacterial colonization and to prevent the growth of remaining bacteria, researchers incorporated various antimicrobial agents or monomers, such as chlorhexidine, quaternary ammonium monomers and silver particles, in adhesive systems^14–19^. Although effective antibacterial capability has been achieved, some agents or monomers may compromise the physico-chemical properties of adhesives^10, 13, 20^.

Poor mechanical properties induced by the degradation of the hybrid layer at the adhesive–dentin interface contribute to the poor longevity of composite restoration^21, 22^. As a consequence of incomplete adhesive infiltration, collagen fibres become exposed and susceptible to degradation by enzymes, especially host-derived matrix metalloproteinases (MMPs)^11, 23^. The latter can be activated during bonding or ageing through different mechanisms^24^. Cysteine cathepsins also exist as exopeptidases in normal and carious dentin and participate in extracellular matrix degradation involving the breakdown of collagen fibre^25^. Therefore, MMP inhibitor and collagen crosslinker are separately or jointly incorporated into adhesives to prevent hybrid layer degradation^26–28^.

However, the achievement of a single function or excessive amounts of additives may not be a good idea because of the ongoing trend existing among manufacturers and researchers to continue simplifying fast, sensitive and universal bonding technology^29^. Thus, one-pot incorporation of a bioactive agent possessing multiple functions, such as simultaneously preventing secondary caries and adhesive interface degradation, is a very promising approach.

**Figure 1:**
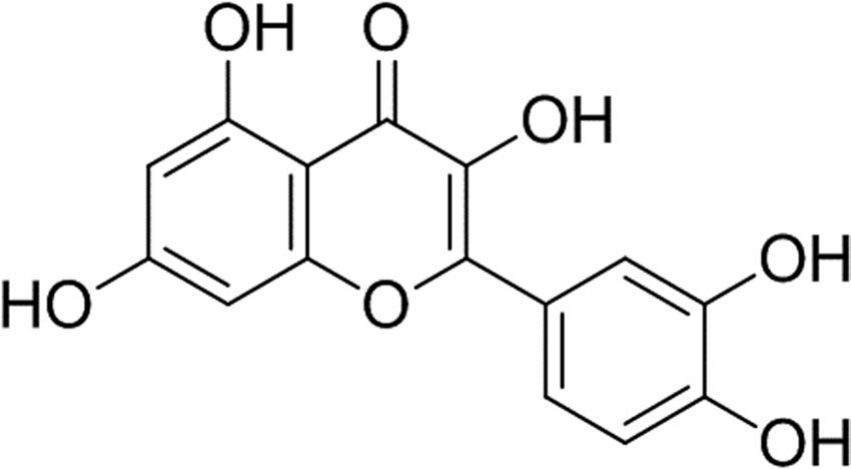
The chemical structure of quercetin.

Naturally derived collagen crosslinkers with high antibacterial abilities for dentin bonding have been widely explored^30–32^. Quercetin (Fig. 1), which is the most common flavonol in the diet^33^, possesses multiple functions, including antioxidative^34^, anticarcinogenic^35^, anti-inflammatory^36^, anti-aggregatory^37^ and vasodilating effects^38^. Quercetin downregulates MMP 2 and 9 protein expression in prostate cancer cells^39^ and elicits significant antibacterial effects on Gram-positive and Gram-negative bacteria^40, 41^. In this case, therapeutic adhesives with multiple functions can be produced through an all-in-one packaging incorporation of quercetin. However, related information on adhesive modification has yet to be obtained.

This study aimed to evaluate the antibacterial and bonding properties of a dental adhesive doped with quercetin at different concentrations. We hypothesised that^1^ the antibacterial performances of unmodified-adhesive and quercetin-doped adhesives do not significantly differ and^2^ quercetin incorporation does not affect bonding properties even after collagenase ageing occurs.

## Results

### Antibacterial ability evaluation by confocal laser scanning microscopy (CLSM) and XTT

**Figure 2:**
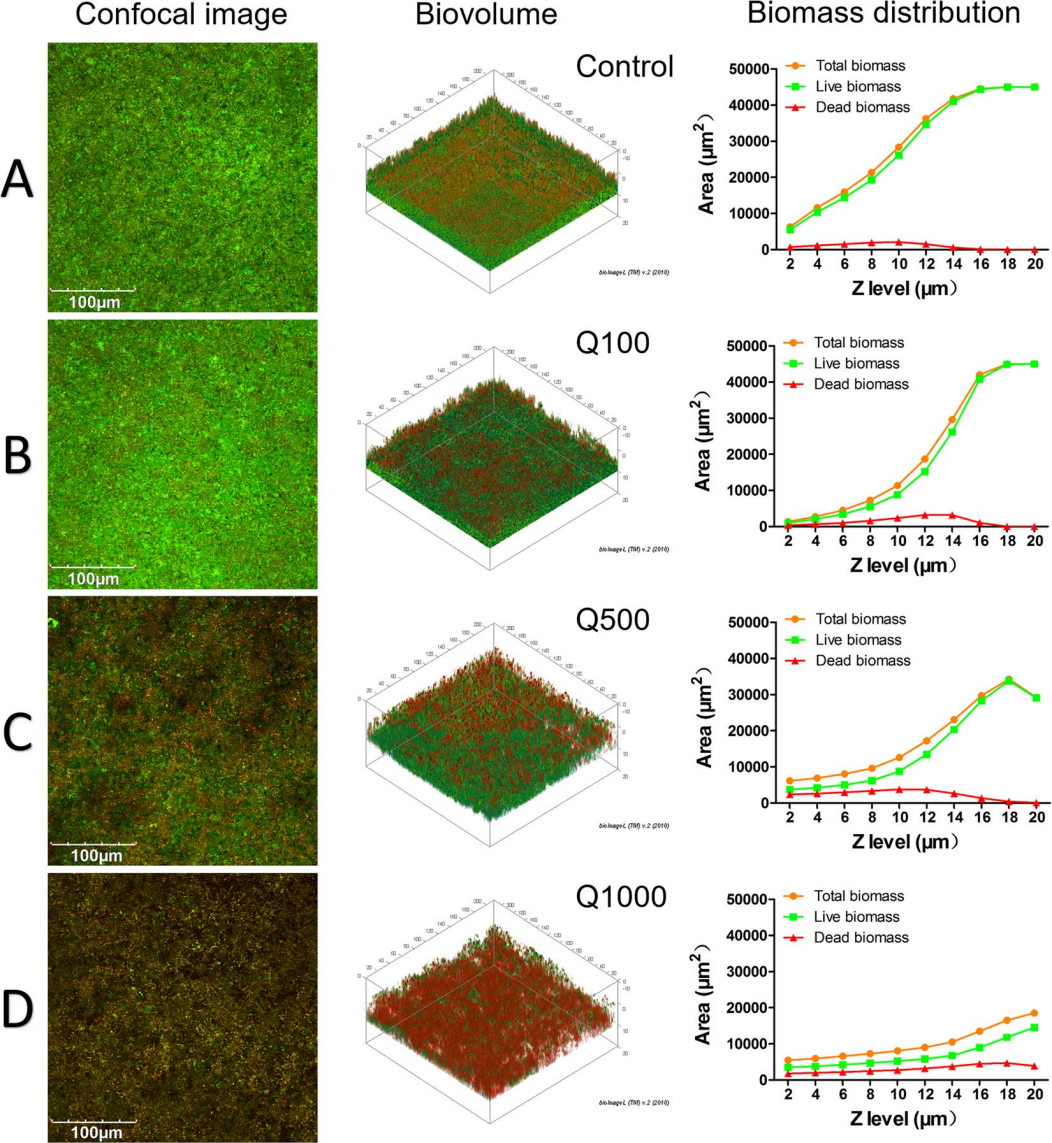
Confocal laser scanning microscopy evaluation of *S. mutans* biofilms grown on adhesives of 4 groups (live-green; dead-red). (**A**) Control group; Quercetin-doped adhesive groups at the concentration of (**B**) 100 ug/mL, (**C**) 500 ug/mL and (**D**) 1000 ug/mL. (Left) Top-down view; (Middle) 2D overlay projections; (Right) corresponding biomass distribution of total, live and dead bacteria at each layer along the Z stack (layer interval = 2 um, scanning depth = 20 um).

Figure 2 shows representative stacked confocal images of *S. mutans* biofilm on different adhesive surfaces, while the 2D overlay images of 10 layers was reconstituted, and the relative distribution of live/dead bacteria biomass at each layer of a Z-stack are also summarized in the line pots. From these information, green dominated the staining in the control group, indicating that the bacteria were primarily alive. The dead bacterial (red spot) distribution in the total biomass gradually increased from the control group to Q100, Q500 and Q1000 adhesive groups. The control group demonstrated the largest total biomass area at each layer of a Z-stack. By comparison, the total biomass at the same layer decreased as the volume of quercetin increased.

**Figure 3:**
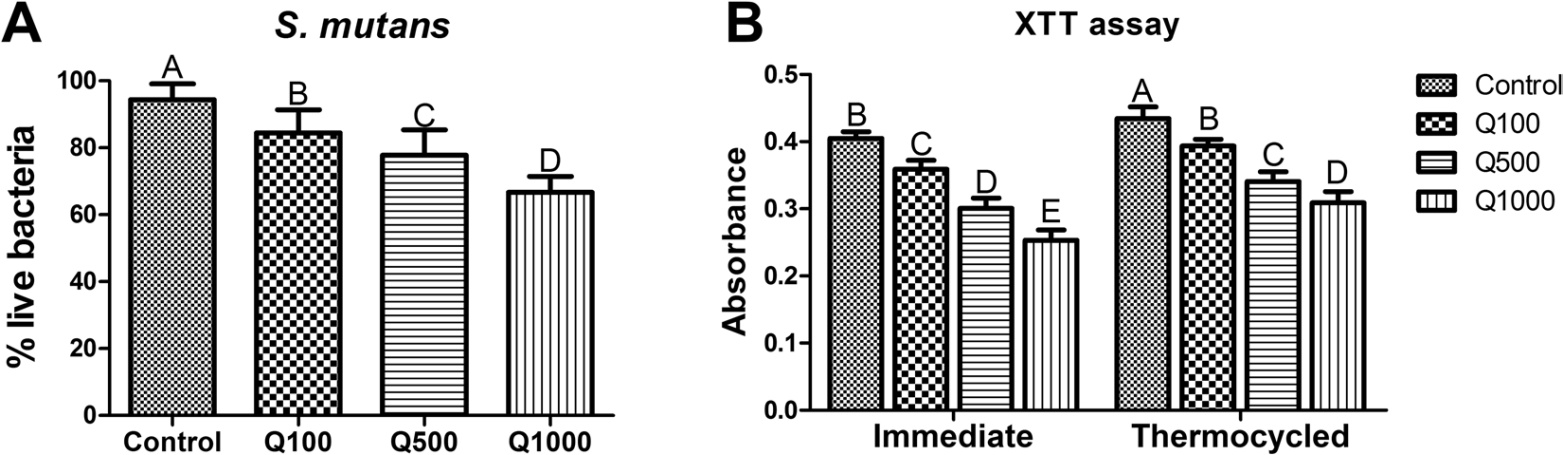
(**A**) Percentage distributions of live bacteria among total biomass based on the CLSM calculation result, n = 10. (**B**) The average XTT OD_570_ values after 24 h incubation of *S. mutans* on adhesives of 8 groups (4 for immediate groups, and 4 for thermocycling aged groups), n = 9. The data are expressed as the mean ± SD, groups with the same superscripts are not statistically significant (*P* > 0.05).

The live bacterial percentage of *S. mutans* biofilm from each group is shown in Fig. 3A. The quercetin-doped adhesive groups exhibited much less live bacteria than the control group (*P* < 0.05). XTT results from Fig. 3B showed that the metabolic activity of *S. mutans* decreased with the increasing of quercetin, especially in the Q500 and Q1000 groups; Although the antibacterial ability of all groups has decreased to some extent after thermocycling, the Q500 and Q1000 groups remained effective in bactericidal ability compared with the immediate control group.

### Degree of conversion (DC) and polymerization rate

**Figure 4:**
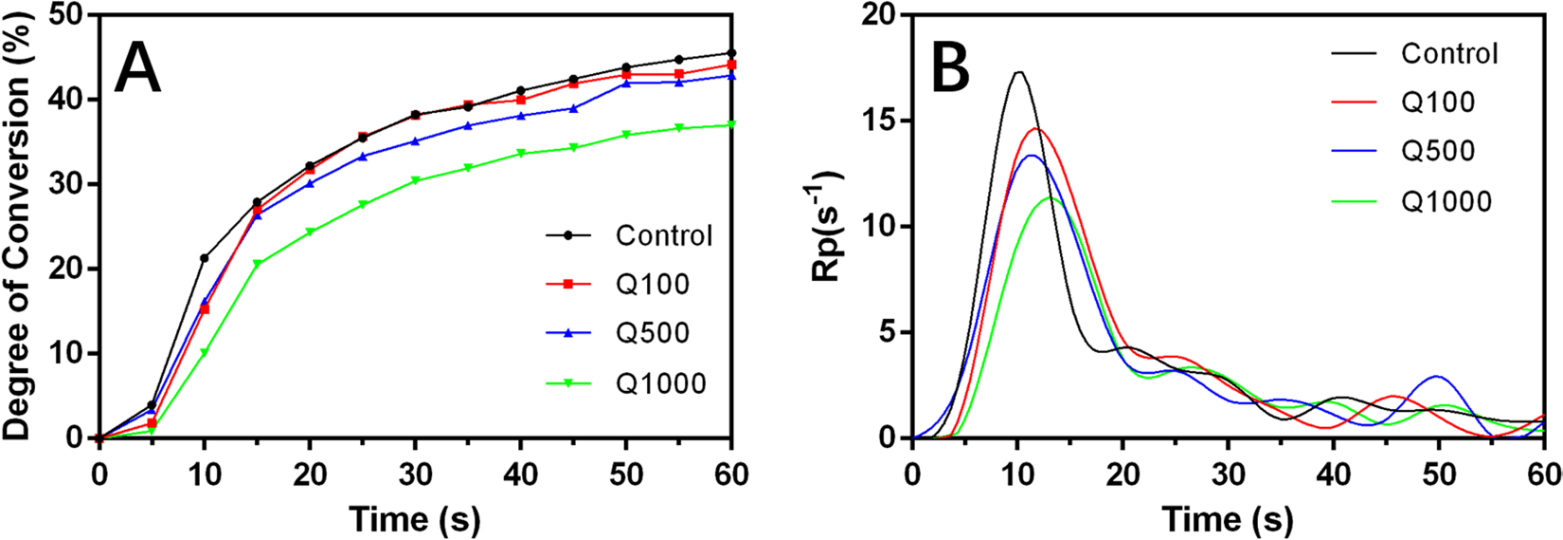
The degree of conversion (**A**) and rate of polymerization (**B**) of 4 adhesive groups.

Figure 4A shows that the DC values of four different adhesive groups exhibited the same tendency of changes with time supplement. After quercetin is incorporated at low concentration (Q100 and Q500), the DC of dentin adhesives remained nearly unchanged compared with the control group. However, the DC value showed an obvious decrease in the Q1000 group.

Figure 4B shows the polymerization rate of four experimental adhesives. Similar waveforms were observed although the position of representative peak expressed an extended tendency with the incorporation of quercetin.

### Microtensile bond strength (MTBS) and Failure modes

Mean MTBS values (expressed in MPa) are calculated and shown in Table 1. All data analyzed showed normality of data and homogeneous variance (*P* > 0.05). Two-way ANOVA showed that the variables both incorporating the volume of quercetin (*F* = 17.069, *P* = 0.000) and collagenase ageing (*F* = 131.381, *P* = 0.000) significantly affected bond strength.

**Table 1:** Microtensile bond strength (MTBS) and failure frequency of each group.

Groups	Aging	MTBS (MPa)	Failure frequency (%)			
			A	CC	CD	M
Control	Immediate	42.29 ± 6.71^a^	90	0	0	10
	Aged	27.86 ± 6.25^d^	50	0	12.5	37.5
Q100	Immediate	42.77 ± 7.80^a^	100	0	0	0
	Aged	27.63 ± 6.86^d^	57.5	0	7.5	35
Q500	Immediate	41.91 ± 7.98^a^	55	0	15	30
	Aged	39.62 ± 6.95^ab^	45	0	7.5	47.5
Q1000	Immediate	35.42 ± 6.15^bc^	75	0	0	25
	Aged	30.95 ± 7.75^cd^	35	7.5	7.5	50

The MTBS values are expressed as mean ± SD in MPa, n = 40. Groups with the same letters are not statistically different (*P* > 0.05). A, adhesive failure; CC, cohesive failure in composite; CD, cohesive failure in dentin; M, mixed failure.

Before ageing, no significant difference on MTBS was found in the Q100 and Q500 groups compared with the control group (*P* > 0.05). However, a low bond strength was obtained when the added amount of quercetin was 1000 µg/mL (*P* < 0.05). After collagenase ageing occurred, the MTBS of the control and Q100 groups significantly decreased (*P* < 0.05). By contrast, the MTBS of the two other groups remained to some extent. Among the collagenase-aged groups, the Q500 group obtained the highest MTBS.

**Figure 5:**
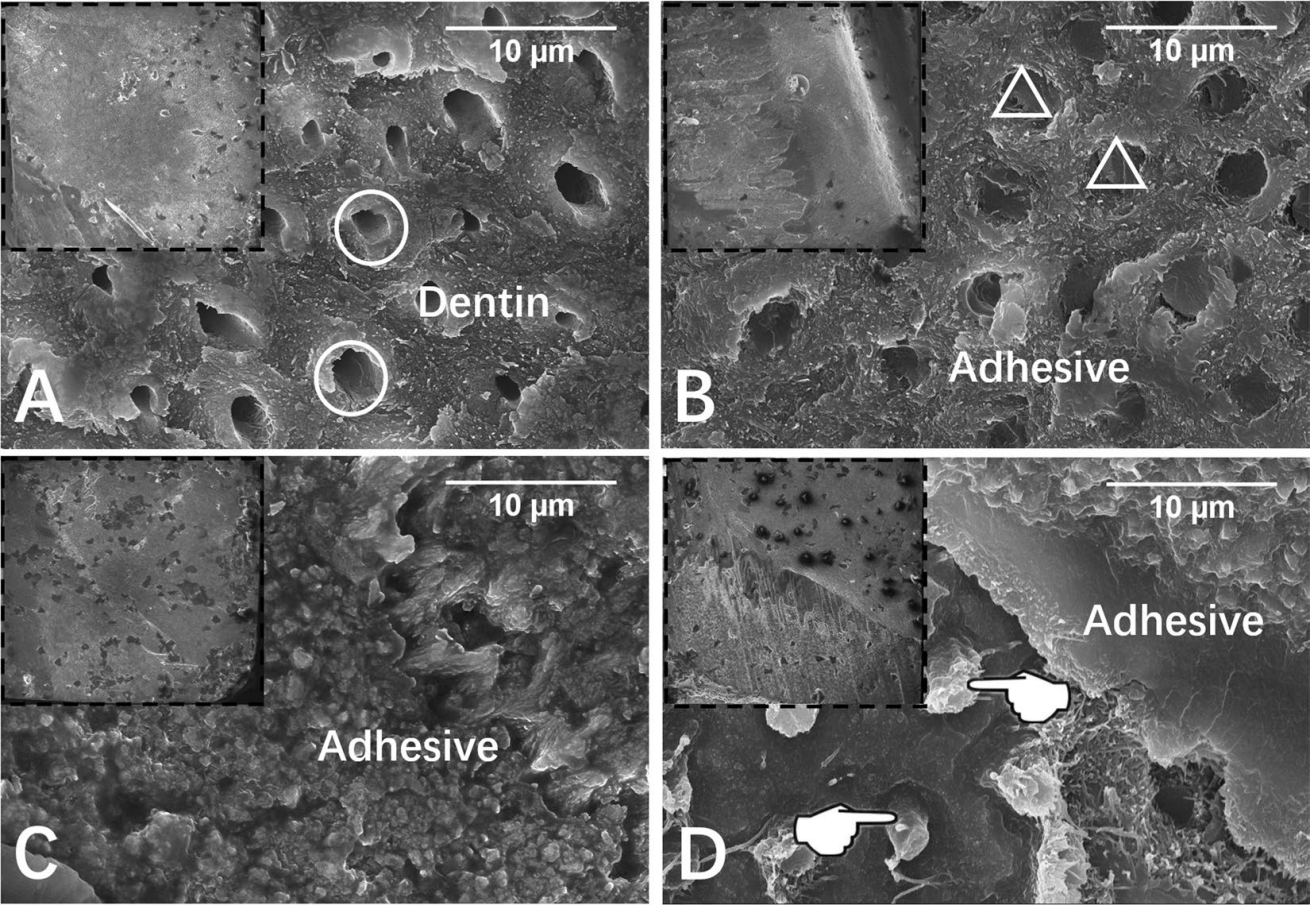
Representative FESEM images (3000×) of fractured dentin sides after microtensile bond strength test. The insert indicates general condition of fractured surfaces (80×). (**A**) Cohesive failure in dentine, from collagenase-aged control group; (**B**) cohesive failure in composite, from collagenase-aged quercetin-doped (1000 ug/mL) group; (**C**) adhesive failure, from immediate quercetin-doped (100 ug/mL) group; (**D**) mixed failure, from immediate quercetin-doped (500 ug/mL) group. Circle: open dentin tubules; triangle: sealed dentin tubules; pointer: resin tags.

The failure frequency distribution is shown in Table 1. Adhesive failure dominated the main failure modes in the immediate groups regardless of quercetin addition. However, mixed failure increased to some extent after collagenase ageing occurred. The representative field emission scanning electron microscopy (FESEM) images are shown in Fig. 5.

### Nanoleakage expression

**Figure 6:**
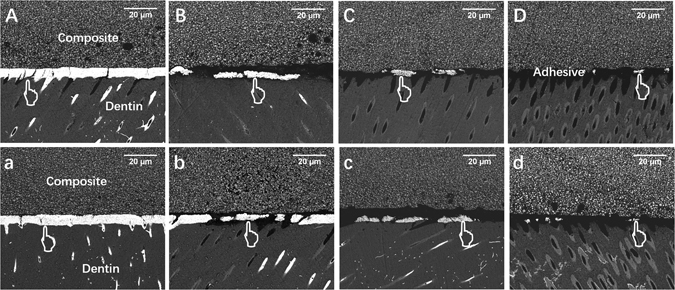
Representative FESEM images (3000×) of nanoleakage expression from different groups. Bonded interface of the immediate groups (**A–D**) and one-month collagenase-aged groups (a–d): (**A**,a) control group; quercetin-doped adhesive groups at the concentration of (**B**,b) 100 ug/mL, (**C**,c) 500 ug/mL and (**D**,d) 1000 ug/mL. Pointer: silver uptake.

Statistical results of nanoleakage expression are shown in Table 2. The Kruskal–Wallis test showed that quercetin-doped adhesive groups possessed lower nanoleakage than the unmodified group (*P* < 0.05) irrespective of collagenase ageing. Meanwhile, a dose-dependent effect of quercetin incorporation on nanoleakage expression was achieved. In other words, nanoleakage expression decreased gradually with the increasing volume of quercetin. The representative FESEM images of nanoleakage are shown in Fig. 6. White metallic silver was distinct along the interface, sometimes filled into dentinal tubules especially in the control group (Fig. 6A,a). The control group and Q1000 separately achieved the highest and lowest nanoleakage expression. The former showed continuous, thick silver penetration (Fig. 6A,a), whereas the latter demonstrated interrupted, sparse distribution (Fig. 6D,d).

**Table 2:** Percentage distribution of nanoleakage scores from each group.

Groups	Time	Nanoleakage expression		
		Score 0–4	%	Statistical difference
Control	Immediate	0	0	A
		1	15	
		2	15	
		3	10	
		4	60	
	Aged	0	0	A, B
		1	15	
		2	20	
		3	30	
		4	35	
Q100	Immediate	0	0	B, C
		1	0	
		2	85	
		3	10	
		4	5	
	Aged	0	0	B, C, D
		1	15	
		2	60	
		3	15	
		4	10	
Q500	Immediate	0	20	C, D, E
		1	30	
		2	15	
		3	20	
		4	15	
	Aged	0	0	B, C, D
		1	35	
		2	35	
		3	15	
		4	15	
Q1000	Immediate	0	10	E
		1	70	
		2	20	
		3	0	
		4	0	
	Aged	0	0	D, E
		1	60	
		2	30	
		3	10	
		4	0	

Kruskal–Wallis test with Dunnett’s post-hoc test. Groups with the same letters are not statistically different (*P* > 0.05), n = 20.

### *In situ* zymography of the hybrid layer

**Figure 7:**
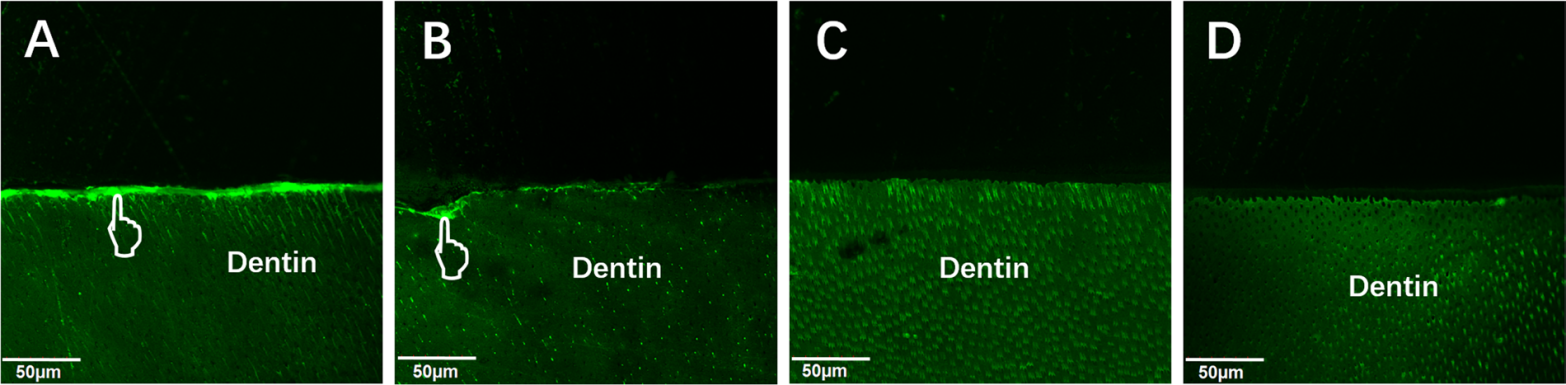
Confocal laser scanning microscopy images of *in situ* zymography labeled with quenched fluorescein-conjugated gelatin. (**A**) Control group; Quercetin-doped adhesive groups at the concentration of (**B**) 100 ug/mL, (**C**) 500 ug/mL and (**D**) 1000 ug/mL. Pointer: indicative of MMP activity.

The representative CLSM images of *in situ* zymography from different groups are shown in Fig. 7. An intense green fluorescence with the hybrid layer was observed in the control group, indicating that the fluorescein-conjugated gelatin was strongly hydrolyzed at these sites (Fig. 7A). When quercetin was incorporated at a concentration of 100 µg/mL, the green fluorescence reduced obviously (Fig. 7B). No intense fluorescence was detected with the hybrid layer in the Q500 and Q1000 groups (Fig. 7C,D).

## Discussion

A versatile adhesive was developed by incorporating quercetin into a commercial dentin adhesive in the present study. The effect of quercetin incorporation on performance of adhesive–dentin restoration was evaluated. The CLSM evaluation and XTT test demonstrated that the antibacterial activity of dentin adhesives was enhanced with rising addition of quercetin. The MTBS results showed that quercetin addition did not affect immediate bond strength at a concentration of 500 µg/mL (*P* > 0.05), and that the bonds were mainly preserved after one-month collagenase ageing. Quercetin-doped adhesive groups possessed lower nanoleakage than the unmodified group (*P* < 0.05) irrespective of collagenase ageing. Real-time Fourier transform infrared (FTIR) spectroscopy showed that the DC of dentin adhesive was not affected after quercetin incorporated at 100 and 500 µg/mL. The latter also demonstrated an efficient inhibition of MMP activity, and an acceptable biocompatibility for human gingival fibroblast cells (Supplementary Fig. S1). Therefore, the null hypotheses were rejected.

As the basis of aesthetic restoration, the durability and stability of adhesive–dentin bonds are still a matter of concern^45^. Biofilm formation induced by *S. mutans* is considered as the main factor for secondary caries at adhesive–dentin interface^46^. Living bacteria existing in mature biofilm possess lower metabolism capability and dull sensitivity to antimicrobial agent compared with planktonic bacteria^14^. Thus, effective inhibition of biofilm formation is essential for an antibacterial adhesive^47^. Our previous study showed that quercetin can significantly inhibit the biofilm formation of *S. mutans* and effectively remove the mature biofilm^48^. Inspired by this finding, we proposed that quercetin might be incorporated into adhesive to combat residual bacterial and biofilm formation at the adhesive–dentin interfaces. The commercial adhesive presented the highest metabolic activity of *S. mutans* and the largest amount of biomass in the present study; this result indicates that the unmodified adhesive exhibited normal bacterial growth without antibacterial capability and accumulated more biofilms, which is consistent with previous studies^49–51^. By contrast, the biofilm metabolic activity and total biomass all reduced by degree after quercetin incorporated into adhesive. Although the absorbance of XTT assay from all groups increased to some extent after thermocycling, the Q500 and Q1000 groups remained effective in bactericidal ability comparing with the immediate control group. This finding indicates that the growth of *S. mutans* biofilm can be inhibited with the increase of quercetin content, while quercetin-doped adhesive might remain long-term antibacterial ability. We speculate that, in the *in vivo* application, the quercetin in adhesive could cross-link with dentin collagen, this ability enables quercetin to stay relatively stable within dentin matrix and limits its releasing rate into dental saliva. In this situation, the interaction between quercetin and dentin matrix could be viewed as a sustained-release system, which guarantee long stable releasing of quercetin and long-term antibacterial effect. Further researches are needed to verify our speculation.

The antibacterial mechanism of quercetin has been explained by researchers. Cowan^52^ thought that flavonoids (including quercetin) can form complex with extracellular soluble proteins, and then bind to bacterial walls to suppress a wide range of microorganisms. Mirzoeva^53^ demonstrated that quercetin can uncouple the energy transducing cytoplasmic membrane and inhibit bacterial motility, resulting in an antimicrobial action. In the present study, no significant difference was found on water contact angle between unmodified adhesive and quercetin-doped adhesives (Supplementary Fig. S2), suggesting that, rather than physically attachment-resistance effect of adhesive modification, the inner antibacterial characteristics of quercetin might bear greater responsibility for *S. mutans* biofilm inhibition. Our previous study^48^ further proved that quercetin suppressed the expression of gtfB and gtfC, comD and comE and promoted the expression of luxS. All these genes are involved in the adhesion, growth and maturation of *S. mutans* biofilm^54–56^.

Besides secondary caries, the autodegradation of collagen matrix at the adhesive–dentin interface is another urgent problem that needs to be resolved^57^. The degradation is usually induced by the action of host-derived MMPs (mainly MMP-2 and MMP-9); the latter can be activated by almost all two-step etch-and-rinse and one-step self-etch adhesives^58^. Quercetin has been proved to downregulate MMP-2 and MMP-9 protein expression in prostate cancer cells^39^. Therefore, to test whether the incorporation of quercetin in adhesive can still exhibit ability to silence MMP activity at the hybrid layer and to preserve the bond strength against ageing is necessary.

The present study shows that an intense green fluorescence with the hybrid layer was observed in the control group, indicating that the endogenous dentin MMP-2 and -9 at the hybrid layer might be activated by dentin demineralization and/or exposure of the collagen during bonding^57^. However, no intense fluorescence was detected with the hybrid layer in the quercetin-doped groups (Q500 and Q1000 groups), demonstrating the silence of enzymatic activity by quercetin.

Collagenase from *Clostridium histolyticum* has been reported to cut collagen chains and accelerate the removal of collagen matrix^59, 60^. Performing *in vitro* ageing test to challenge adhesive–dentin bonds was adopted here. The present study shows that the appropriate incorporating content (100 and 500 µg/mL) of quercetin exhibits no significant influence on immediate bond strengths. After collagenase ageing, the MTBS of unmodified adhesive decreased obviously. However, the quercetin-doped specimens preserved the MTBS to some extent, especially at a concentration of 500 µg/mL. These results suggested that quercetin-doped adhesive shows the ability to protect collagen matrix at the adhesive–dentin interfaces from bacterial or proteolytic activity.

Nanoleakage expression is always used to investigate the sealing ability of an adhesive system because nanoholes or canals can serve as pathways of water penetration or bacterial attack, resulting in the failure of dentin bonding^61, 62^. The present study shows that nanoleakage expression decreased with rising content of quercetin in adhesive, irrespective of collagenase ageing. Our results suggested that quercetin-modified adhesive may have a better sealing ability on bonding interface compared with unmodified adhesive. The reasons behind this phenomenon may be attributed to the protective effect of quercetin on dentin collagen;^63^ quercetin molecules may bind within hydrophobic pockets of collagen, and entrapped collagen protein by hydrogen bonds, resulting the crosslinking of collagen and the increase of hydrophobicity^64, 65^. Thus, the formation of water canals at bonding interfaces decreased.

In the present study, quercetin was firstly dissolved into ethanol at different concentrations, followed by incorporation into adhesives at same volume of solution, according our previous experience to minimize the possible negative influence on bonding efficiency and handling property^30^. Real-time FTIR showed that the appropriate incorporation content of quercetin (100 and 500 µg/mL) shows no significant influence on the DC of adhesive. However, the DC decreased with the addition of 1000 µg/mL quercetin into adhesive. This result might be attributed to the free radical scavenging ability of quercetin; too high quercetin incorporation may disturb the formation of linear polymer chains, leading to the uncompleted free radical polymerization of the adhesive^66^. The varying tendencies of DC basically echo the changes of MTBS among different groups, wherein a low MTBS was achieved in the Q1000 immediate group.

During adhesive modification, the desired biocompatibility of additives should be achieved. Synthetic glutaraldehyde as a well-known crosslinking agent has been used to immobilize collagen proteins via intermolecular covalent crosslinking^67^. However, the cytotoxicity of this substance impedes its applications in dentistry^68^. As a potential alternative, quercetin is used because its collagen cross-linking effect is stronger and its cytotoxic concentration is 100 times lower than those of glutaraldehyde^69^. Quercetin also induces the dentinogenic differentiation of dental pulp cells^70^. Modified-dentin adhesives may present different cytotoxicities by the interaction of multiple components^71, 72^. Thus, we identified the cytotoxicity of quercetin-doped adhesive in our study. Our MTT results showed no significant difference in the cell viability of HGFs after quercetin was incorporated at concentrations of 100 and 500 µg/mL (Supplementary Fig. S1). This finding indicated that quercetin is characterised by low cytotoxicity and acceptable biocompatibility for clinical applications.

In our study, a versatile quercetin-doped adhesive was developed, and the effectiveness of quercetin incorporation on the comprehensive performance of adhesive–dentin restoration was evaluated. The quercetin-doped adhesive inhibited the growth of *S. mutans* biofilm, and the bonding properties were mainly preserved after collagenase ageing occurred. Our results confirmed that 500 µg/mL quercetin could be the optimum adding content to reach the balanced status of the antibacterial ability, bonding strength, conversion degree and cytotoxicity for adhesive modification. Thus, a simple, safe and workable strategy was established to produce therapeutic adhesives by incorporating quercetin. Further studies and clinical trials should be performed to test the validity of this strategy against various *in vitro* ageing challenges, such as long-term water storage, thermocycling, NaOCl storage and pH cycling.

## Methods

### Preparation of experimental materials

Quercetin powder (Sigma–Aldrich, St. Louis, MO, USA) was directly dissolved into pure ethanol under water-bath heating at 37 °C to achieve concentrations of 1.0 wt%. A commercially available dental adhesive, Adper^TM^ Single Bond 2 (SB) (3 M ESPE, St. Paul, MN, USA) was used as the parent material. Then the quercetin/ethanol was incorporated into SB at appropriate ratio to get the final concentration of 100 μg/mL (Q100), 500 μg/mL (Q500) and 1000 μg/mL (Q1000) quercetin in the adhesive, respectively. SB without quercetin served as the negative control.

### Bacterial culture and biofilm preparation

*S. mutans* Ingbritt, provided by the School of Stomatology, Wuhan University, was cultured overnight at 37 °C for 24 h in Brain Heart Infusion (BHI) broth (BD, Sparks, MD, USA) anaerobically. Bacterial suspension was adjusted to 1 × 10^8^ CFU/mL for further usage. The cover of a sterile 96-well plate was used as a mold for specimen preparation. Ten microliters of each test material were spread on the bottom of the 96-well cover (four samples for each group), followed by light curing with a light-curing unit (Bluephase Style, Ivoclar-Vivadent Amherst, NY, USA) for 10 s. Composite resin (Charisma; Heraeus Kulzer, Hanau, Germany) was applied above the adhesive layer and photo-cured to obtain specimens of 6.4 mm in diameter and 1 mm in thickness. All specimens were dried at room temperature, and disinfected under ultraviolet light for 1 h on each surface.

Specimens of each group were placed in the wells of a 24-well plate, while the adhesive layer facing upward. A mixture of 10 μL *S. mutans* cell suspension (10^8^ CFU/mL) and 1 mL BHI added with 1% sucrose was injected into each well. After anaerobic culture at 37 °C for 24 h, the biofilm-coated specimens were gently immersed in 1 mL of sterile phosphate buffer solution (PBS) twice to wash away the non-adherent bacteria cells, then transferred to another 24-well plate with the top surface facing upward.

### Live/dead staining of biofilms

The Live/Dead Bacterial Viability Kit (Molecular Probes, Invitrogen, USA) was used to stain biofilm-coated specimen for 15 min (n = 1 for each group). Specifically, the live bacteria could be penetrated by SYTO-9 to emit green fluorescence at 488 nm wavelengths, whereas the dead bacteria with damaged membrane could be stained by propidium iodide (PI) to emit red fluorescence at 568^23^. The specimens were rinsed gently and observed via a confocal laser scanning microscope (CLSM; Fluoview FV1200, Olympus, Tokyo, Japan) at 40 × magnification. A series scanning at a Z-stack of 2 μm was performed to produce ten images from the adhesive surface to the top of the biofilm. BioImageL software (Faculty of Odontology, Malmö University, Malmö, Sweden) was used to produce 2D overlay images of CLSM along the Z-stack, the total biomass and live/dead bacterial distributions at each scanning layer were also analyzed.

### Antibacterial evaluation by XTT assay

In order to evaluate the long-term antibacterial effect of quercetin-doped adhesive, additional specimens were prepared and placed in a thermocycling machine from 5 °C to 55 °C for 10,000 cycles, with the dwell time set at 15 s, to simulate an entire year’s clinical physiological ageing. After that, biofilm-coated specimens from each group was prepared as mentioned above. The XTT/Menadione reagent was prepared by mixing XTT (Sigma, St. Louis, MO, USA) solutions (1 mg/mL) with menadione (Sigma, St. Louis, MO, USA) (1 mM) in the volume ratio of 12.5:1. Three biofilm-coated specimens from each group were transferred into separate centrifuge tubes containing 4 mL of sterile PBS (0.01 mM, pH 7.3), then 54 μL of XTT/Menadione reagent was added and the solutions were mixed gently. After an incubation period of 4 h at 37 °C with light protection, the microtubes were centrifuged at 3000 rpm for 3 min to form bacterial suspension solutions. Three readings of each tube from each group (n = 9 each group) was measured under 492 nm using a spectrophotometer (Powerwave 340, Bio-tek Instruments, Winooski, VT, USA). Since quercetin has intrinsic fluorescence^42^, the background absorbance of each group was subtracted from OD492 value.

### Degree of conversion and polymerization rate

The degree of conversion (DC) and polymerization rate of the experimental adhesives was evaluated in triplicate by real-time Fourier Transform Infrared Spectroscopy (FTIR) equipped with an ATR crystal with a 45-degree mirror angle. The light-curing unit was set at a standardized distance of 5 mm. One drop of each experiment adhesive was spread on the surface of a potassium bromide (KBr) Pellet. The adhesive layer was photo-cured for 20 s, while the FTIR spectrum began to scan at the same time. The scanning rang was set as 1800–1500 cm^−1^ with a resolution of 4 cm^−1^ in the transmittance mode (Nicolet 6700, ThermoFisher, MA, USA), one scan was acquired every 5 s for 60 s after the beginning of light curing which was also performed during the entire 60 s of evaluation. The DC was calculated based on the intensity of the C=C stretching vibrations at 1635 cm^−1^, while the symmetric ring stretching at 1608 cm^−1^ from the polymerized and non-polymerized samples as an internal standard. Curve fitting was plotted using logistic non-linear regression, and the polymerization rate (Rp (S^−1^)) was calculated as the DC at time t substracted from the DC at time t-1. The coefficient of determination was greater than 0.98 for all curves.

### Bonding specimen preparation

A total of 40 caries-free human third molars were used in present study. All teeth were collected after obtaining donors’ informed consents, the protocol utilized in the present study was reviewed and approved [2011(067)] by the Ethics Committee for Human Studies of the School and Hospital of Stomatology, Wuhan University, China. The methods employed were performed in accordance with the approved guidelines and regulations. The teeth were stored in 1% chloramine at 4 °C within one month before use. The teeth were sectioned parallel to the occlusal crown with a low-speed water-cooled diamond saw (Isomet; Buehler, Evanston, IL, USA). The exposed dentin surface was wet-ground with 600-grit SiC paper for 60 s to produce a standardized smear layer. The dentin surface of each specimen was etched with 35% phosphoric-acid gel (3 M ESPE, St.Paul, MN, USA) for 15 s and rinsed with deionized water thoroughly. Then, one of four experimental adhesives (control, Q100, Q500 or Q1000) was applied on the blotted water-moist dentin surface, followed by gently agitated for 10 s and air stream for another 10 s. The adhesive was light-cured for 20 s, and 4 mm-thickness of resin composite (Charisma, Haraeus Kulzer, Hanau, Germany) were built up at 1 mm intervals with 20 s light curing each. There are 10 bonded tooth in each group.

### Microtensile bond strength (MTBS) test

After storing in deionized water at 37 °C for 24 h, the bonded teeth were sectioned perpendicular to the bonding interfaces to produce slabs of 0.9 mm thickness. Six middle slabs from each group were left for nanoleakage evaluation (n = 4) and *in situ* zymography of the hybrid layer (n = 2), other slabs were further sectioned vertically to produce beams with a dimension of 0.9 mm × 0.9 mm. Four qualified beams from each tooth (n = 40 each subgroup) were immediately subjected to MTBS testing, and the other four (n = 40) were tested after one-month collagenase aging. The collagenase solution was prepared by dissolving bacterial (*Clostridium histolyticum*) collagenase (Sigma–Aldrich, St. Louis, MO, USA) into artificial saliva to obtain a concentration of 0.1 mg/mL. The aged specimens were immersed in the 0.1 mg/mL collagenase-containing artificial saliva in the dark at 37 °C, replaced every 3 days.

The prepared beam was attached to a MTBS tester (Bisco Inc., Schaumburg, IL, USA) with a cyanoacrylate glue (Zapit, Dental Ventures of America, Corona, CA). Each beam was loaded in tension until failure at a cross-head speed of 1 mm/min. The dimension of each beam was measured by a digital calliper.

After MTBS test, the fractured dentin specimens were dehydrated, sputter-coated with gold, and then observed via field-emission scanning electron microscopy (FESEM; Sigma, Zeiss, Germany). The failure modes were classified into four groups:^43^ (A) adhesive failure; (CD) cohesive failure in dentin; (CC) cohesive failure in composite; and (M) mixed failure.

### Interfacial nanoleakage evaluation

The four middle slabs from each group were randomly assigned to prepare nanoleakage specimen immediately or after one-month collagenase aging (n = 2 each subgroup). The slabs were coated with two layers of nail varnish, leaving 1-mm-width area from the bonded interface. The slabs were then immersed in 50 wt% ammoniacal AgNO_3_ solution (pH 9.5) in the dark for 24 h, and then in a photo-developing solution for 8 h under a fluorescent light irradiation. All slabs were wet-polished with 600-, 1200-, 2000-, and 5000-grit SiC papers and 0.25 um diamond paste using a polishing cloth. Specimens were then clean, dried, sputter-coated with carbon, and observed under FESEM. Ten fields-of-view along the bonding interface of each slab were randomly captured (n = 20 each subgroup). Image J (NIH, Frederick, MD, USA) was utilized to calculate the percentage of silver uptake along the bonding interface, recorded by two obervers as follows:^44^ 0, no nanoleakage; 1, < 25% nanoleakage; 2, 25% ≤ 50% nanoleakage; 3, 50% ≤ 75% nanoleakage; and 4, >75% nanoleakage. Inter-observer agreement was measured by the Kappa test (K = 0.85).

### *In situ* zymography of the hybrid layer

The remaining two bonded slabs from control, Q100, Q500 and Q1000 groups were prepared to evaluate *in situ* zymography of the hybrid layer. One drop (50 μL) of the quenched fluorescein-conjugated gelatin mixture (E-12055, Molecular Probes, Eugene, OR, USA) was placed on top of each slab. The slabs were placed on glass slides and covered by coverslips, incubated in humidified chambers at 37 °C for 24 h with light protection. The amount of green fluorescence, indicative of MMP activity, was observed under CLSM (Fluoview FV1200, Olympus, Tokyo, Japan) with an excitation of 488 nm and an emission of 530 nm.

### Statistical analysis

The MTBS test results were analyzed using Two-Way (variables: the incorporating volume of quercetin and collagenase aging or not) analysis of variance (ANOVA) and post-hoc Tukey’s test. Statistical differences between the scores of the nanoleakage groups were analyzed using the Kruskal–Wallis with Dunnett’s post-hoc test while inter-examiner reliability was assessed using the Cohen’s kappa test. Live bacteria distributions, MTT and XTT assay results were analyzed using One-Way analysis of variance (ANOVA) followed by post-hoc Tukey’s test. All statistical analyses were performed using SPSS (IBM SPSS Statistics 20, Armonk, NY, USA). The significance level was set at 0.05 for all tests.

## Data Availability

All data generated or analyzed during this study are included in this published article, while cytotoxicity evaluation and water contact angle measurement was shown in Supplementary Information files.

## Electronic supplementary material

Supplementary Information
