# Supplementary material for: High-performance therapeutic quercetin-doped adhesive for adhesive–dentin interfaces
Source: Sci Rep. 2017 Aug 15;7:8189. doi: 10.1038/s41598-017-08633-3 (PMC5558009; doi:10.1038/s41598-017-08633-3)
Supplement: Supplementary file 1 — Supplementary Information [file 41598_2017_8633_MOESM1_ESM.pdf]

Supplementary Information (Scientific Reports)

High-performance therapeutic quercetin-doped adhesive for adhesive–dentin interfaces

Hongye Yang [1], Kang Li [1], Huiyi Yan, Siying Liu, Yake Wang*, Cui Huang *

The State Key Laboratory Breeding Base of Basic Science of Stomatology (Hubei-MOST) & Key Laboratory for Oral Biomedical Ministry of Education, School & Hospital of Stomatology, Wuhan University, Wuhan, People's Republic of China

[1] These authors contributed equally to this work

*Corresponding author: Cui Huang &Yake Wang. The State Key Laboratory Breeding Base of Basic Science of Stomatology (Hubei-MOST) & Key Laboratory for Oral Biomedical Ministry of Education, School & Hospital of Stomatology, Wuhan University, Wuhan, People's Republic of China

E-mail address: huangcui@whu.edu.cn; yake416@163.com.

1. Methods

1.1 Cytotoxicity evaluation with MTT assay

Human gingival fibroblast cells (HGFs) were cultured in α-modified essential medium (α-MEM) (Hyclone, Thermo Fisher Scientific Inc, Waltham, MA, USA) supplemented with 10% fetal bovine serum (FBS) (Hyclone, Thermo Fisher Scientific Inc) at 37°C with 5% CO_2 in a humidified environment. The HGFs used for experiments were chosen from third passage.

Five groups were selected for cell viability assessment, namely 10% FBS-supplemented α-MEM group (blank control), unmodified group, Q100, Q500, and Q1000 group. Ten microliter experimental adhesives were added into 15 mL centrifuge tube respectively, centrifuged 15 s until the adhesive was at the bottom of tube, and light cured 20 s with LED light. Ten milliliter double-distilled water were added into the centrifuge tube and stored at 37% for 24 h. After that, the distilled water was pipetted out and 10 mL α- MEM with 10% FBS was added in the centrifuge tube. The four tubes were cultured at 37°C with 5% CO_2 for 24 h, and then the storage medium was used for cell seeding.

The HGFs were added into a 96-well plate with density of 5,000 cells per well, and cultured at 37°C with 5% CO_2 for 24 h. Then the old medium was pipetted out, with the five groups' medium added respectively. After 24 h culturing, 10 μL MTT (5 mg/mL) assay was added into each well and incubated for 4 h, then the medium was removed, and 200 μL DMSO solution was added. Finally, the optical density was measured at 490 nm and background absorbance was subtracted. The results were expressed as the relative cell viability (%) compared with blank control group (10% FBS-supplemented α-MEM). The experiment was performed in quintuplicate.

1.2 Water contact angle evaluation

One hundred microliter of each experimental adhesives was dropped on the microscope slide. After fully dispersion on the slide, the adhesives were polymerized with LED light for 20 s. The water contact angles were measured by putting a drop of deionized water (5 μL) on the surface of polymerized adhesives at first, then the Dataphysics OCA 20 instrument (Dataphysics, Germany) was used to detect the angles between water and polymerized

adhesives at the point of intersection. The experiment was performed in quintuplicate.

2. Results

2.1 Cytotoxicity evaluation with MTT assay

All bonding adhesives were tested on human gingival fibroblasts (HGFs) and evaluated for cytotoxicity by MTT assay after 24 h incubation (Fig. S1). The commercial adhesive (control) presented an acceptable cytotoxicity compared with the blank group (P>0.05). Furthermore, no significant difference in cell viability was observed in the Q100 and Q500 groups compared with the control group (P>0.05). However, strong cytotoxicity was observed in the Q1000 group (P<0.05). This result indicates that quercetin incorporation exhibits a dose-dependent cytotoxicity effect on HGFs.

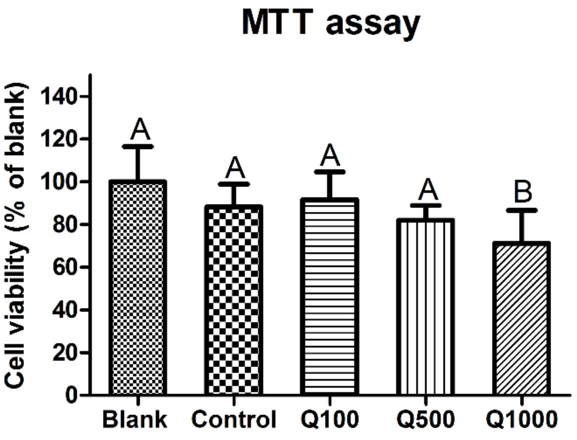

Figure S1. Evaluation of human gingival fibroblast cell viability of exposed to different experimental adhesives. The data are expressed as the mean ± SD, n=5. groups with the same superscripts are not statistically significant (P > 0.05).

2.2 Water contact angle evaluation

The water contact angle and respective views are shown in Fig. S2. The average contact angle (Mean ± SD) for the Control, Q100, Q500, and Q1000 groups were 36.73° ± 3.69°, 32.98° ± 2.40°, 34.14° ± 2.15° and 35.37° ±3.38°, respectively. The values of water contact angle of all the groups have no significant difference (P > 0.05).

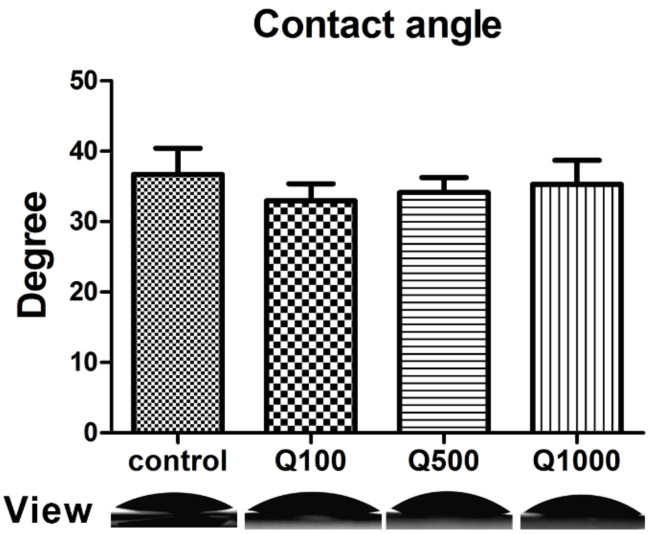

Figure S2. The degree of water contact angle and respective views of the four experimental groups. The data are expressed as the mean ± SD, n=5. All the groups have no significant difference ($P > 0.05$).
